# Whole-Body MRI Is an Effective Imaging Modality for Hematological Malignancy Treatment Response Assessment: A Systematic Review and Meta-Analysis

**DOI:** 10.3389/fonc.2022.827777

**Published:** 2022-02-18

**Authors:** Guisen Lin, Xiaodan Zong, Yaowen Li, Weiting Tan, Weisheng Sun, Siqi Zhang, Yungen Gan, Hongwu Zeng

**Affiliations:** ^1^ Department of Radiology, Shenzhen Children’s Hospital, Shenzhen, China; ^2^ Department of Radiology, The Third Affiliated Hospital, Sun Yat-sen University (SYSU), Guangzhou, China; ^3^ China Medical University, Shenyang, China; ^4^ Shantou University Medical College, Shantou, China

**Keywords:** whole-body MRI, diagnostic value, treatment response assessment, hematological malignancies, meta-analysis, lymphoma, multiple myeloma, sarcoma

## Abstract

**Objectives:**

To evaluate the diagnostic accuracy of whole-body MRI (WB-MRI) for assessment of hematological malignancies’ therapeutic response.

**Methods:**

PubMed, Embase, and Web of Science were searched up to August 2021 to identify studies reporting the diagnostic performance of WB-MRI for the assessment of hematological malignancies’ treatment response. A bivariate random-effects model was applied for the generation of the pooled diagnostic performance.

**Results:**

Fourteen studies with 457 patients with lymphoma, multiple myeloma, and sarcoma (very small proportion) were analyzed. Overall pooled sensitivity and specificity of WB-MRI were 0.88 (95% CI: 0.73–0.95) and 0.86 (95% CI: 0.73–0.93), respectively. Studies using whole-body diffusion-weighted imaging (WB-DWI) showed higher sensitivity than those that did not (0.94 vs. 0.55, p = 0.02). The pooled concordance rate of WB-MRI to assess hematological malignancies’ treatment response with reference standard was 0.78 (95% CI: 0.59–0.96). WB-MRI and PET/CT showed similar diagnostic performance (sensitivity [0.83 vs. 0.92, p = 0.11] and specificity [0.87 vs. 0.76, p = 0.73]).

**Conclusion:**

WB-MRI has high diagnostic performance for hematological malignancies’ treatment response assessment. The adding of WB-DWI is strongly associated with increased sensitivity.

## Introduction

Assessment of cancer treatment effectiveness is the basis for treatment plan adjustment, which provides guidance for clinicians on whether to continue or change treatment strategies. Therapeutic response assessed by imaging is particularly crucial for patients who undergo non-surgical treatment since histological confirmation of the tumor regions is not available. For hematological malignancies that are disseminated, monitoring the treatment response of the whole body is of utmost importance. Currently, this requires not only assessing anatomically changes of tumor size but also monitoring metabolic changes, which could be provided by ^18^F-FDG PET/CT before and after treatment (1).

With recent technological advances and the advantage of zero radiation exposure, whole-body MRI (WB-MRI) has been increasingly used as a clinical tool for cancer staging (2) and screening (3). Moreover, WB-MRI has shown great potential for assessing the therapeutic response of cancer (4). Compared with PET/CT that provides metabolic information of tumors, WB-MRI can detect restricted proton diffusion when combined with whole-body diffusion-weighted imaging (WB-DWI) (5).

However, controversial results remain for WB-MRI as a surrogate imaging modality for hematological malignancies’ treatment response assessment. Some scholars reported that WB-MRI had comparable results for treatment response assessment with PET/CT (6), while others reported an inferior performance compared to PET/CT (7). The correlation between tumor metabolism changes and diffusion was also controversial, with some investigators reporting a positive correlation with PET/CT (8) and others reporting a mismatch result (9). Therefore, the aim of the present study was to perform a systematic review and meta-analysis to evaluate the diagnostic value of WB-MRI for the assessment of hematological malignancies’ treatment response.

## Materials and Methods

### Literature Search

PubMed, Embase, and Web of Science were searched to identify studies exploring the diagnostic value of WB-MRI for cancer treatment response. The search was focused on the following phases: “whole-body magnetic resonance imaging”, “Whole-body MRI”, “WB-MRI”, “Whole-body diffusion weighted imaging”, “Whole-body DWI”, “WB-DWI”, “treatment response”, “therapeutic response”, and “response assessment”. The search process terminated in August 2021. The detailed search strategy is provided in the **Supplementary Material**.

### Study Selection

Two reviewers (GL and XZ) independently screened the abstracts of the identified articles. Disagreement was resolved by consulting a third reviewer (YG or HZ). Full text of the articles would be subsequently reviewed if they fulfilled the following criteria: 1) treatment response assessment of any type of hematological malignancies using WB-MRI with or without WB-DWI; and 2) article was written in English. The exclusion criteria of the articles with full text reviewed were as follows: 1) study with data insufficient to calculate true positive (TP), true negative (TN), false positive (FP), and false negative (FN) for construction of a 2 × 2 contingency table; 2) assessment of treatment response using automatic methods or texture analysis; 3) assessment of metastatic cancers without assessing primary cancer; and 4) treatment response assessment of previously treated cancer. Related citations in eligible articles were assessed for inclusion.

### Data Extraction and Quality Assessment

Two reviewers (WS and SZ) independently extracted data of all included studies with any disagreement resolved through discussion. For all the included studies, authors, year of publication, sample size, mean age of patients, reference standard for treatment response, type of cancers, application of WB-DWI or not, application of contrast enhancement or not, study design (prospective or retrospective), MRI field strength, and data for the diagnostic value of WB-MRI to assess treatment response were extracted. We defined patients with complete response (CR), stringent CR, very good partial response (PR), and PR as a response to treatment, while progressive disease (PD) and stable disease (SD) were defined as treatment failure. For the construction of the 2 × 2 contingency table, TP was defined as the true prediction of response to treatment, FP was defined as the false prediction of response to treatment, TN was defined as the true prediction of treatment failure, and FN was defined as the false prediction of treatment failure. The TP, TN, FP, and FN for PET/CT were also extracted if the study used non-PET/CT-based criteria as the reference standard. For studies that reported the treatment response as CR, PR, SD, and PD, the concordance rate of WB-DWI for assessment was calculated, which was defined as the number of true predictions of CR, PR, SD, and PD using WB-MRI, divided by the total number of CR, PR, SD, and PD using the reference standard.

Quality Assessment of Diagnostic Accuracy Studies (QUADAS-2) was used to evaluate the quality of the included studies (10). Two reviewers independently evaluated the quality of the studies with any disagreement resolved with consensus.

### Statistical Analysis

All statistical analyses were conducted using STATA version 14.0 (StataCorp, College Station, TX, USA). The pooled sensitivity, specificity of WB-MRI to assess therapeutic response, and the pooled concordance rate of WB-MRI were calculated using a random-effects model (11). The calculation was conducted on a per-patient follow-up basis. The summary receiver operating characteristic curves (SROCs) and the area under the SROCs were generated for evaluating the diagnostic performance. Heterogeneity was assessed by I^2^. The possible cause of heterogeneity was analyzed using meta-regression. Univariate meta-regression was conducted initially. If more than one factor was identified to be associated with the heterogeneity through univariate meta-regression, multivariate meta-regression including all the identified factors was performed subsequently. Multilevel mixed-effects logistic regression was used to compare the diagnostic performance of WB-MRI with PET/CT for assessing treatment response with a significant level of p < 0.05. Publication bias was assessed using Deek’s funnel plot.

## Results

### Literature Search and Study Selection

**Figure 1:**
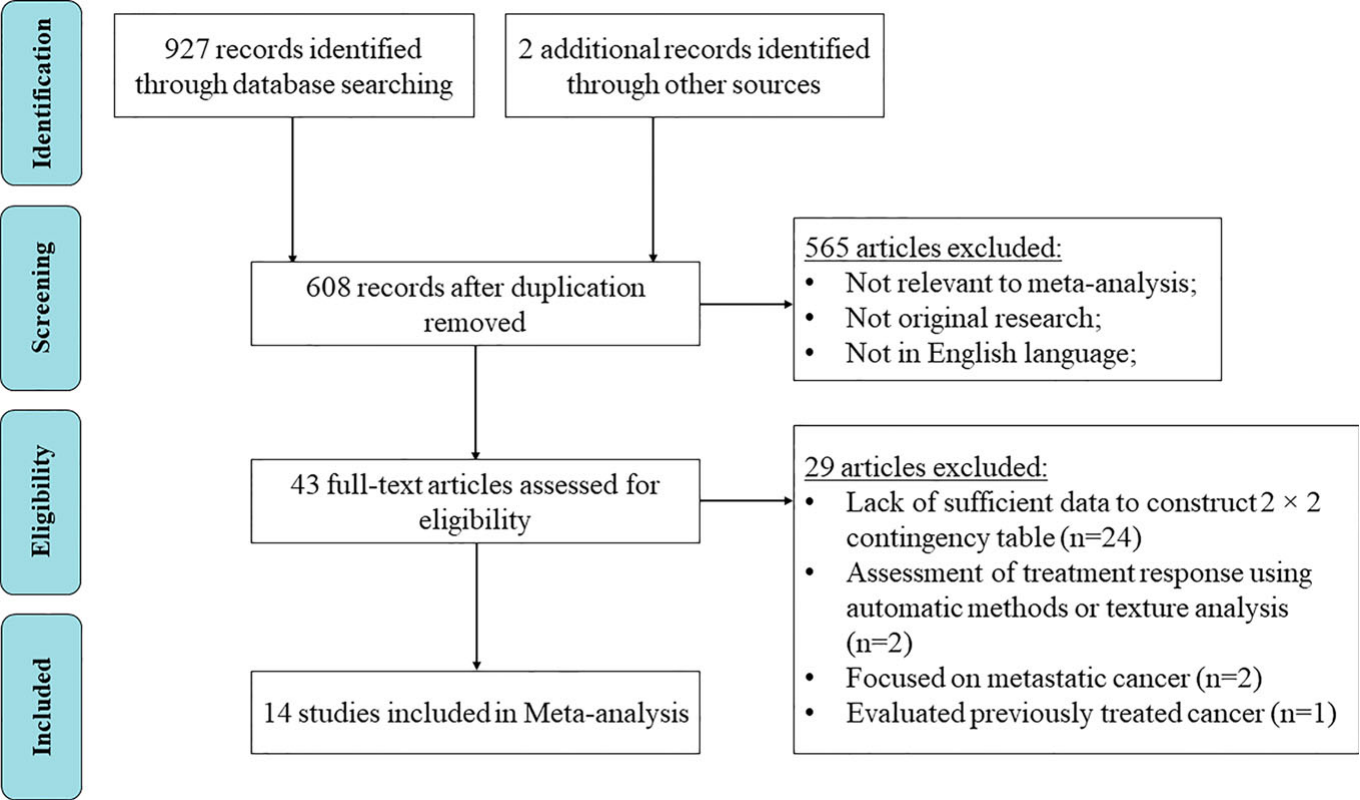
Preferred Reporting Items for Systematic Reviews and Meta-Analyses (PRISMA) flow diagram for article identification and inclusion.

A total of 929 articles were identified through initial search (**Figure 1**), of which 469 articles were identified from PubMed, 368 from Embase, 90 from Web of Science, and 2 from the reference list of one identified article. Following duplication removal, 608 articles remained. After reviewing the abstract, 565 articles were excluded. With the remaining 43 articles, 14 studies met the inclusion criteria for the quantitative analysis (12–25).

### Basic Characteristics of the Included Studies

**Table 1** summarizes the basic characteristics of the included 14 studies. The present meta-analysis included a total of 457 patients with lymphoma, multiple myeloma, or sarcoma (a very small portion of cases in one study). The total number of follow-up for assessing treatment response was 562. Eight studies analyzed patients with multiple myeloma, 5 analyzed patients with lymphoma, and 1 analyzed patients with lymphoma and sarcoma. Three studies evaluated the diagnostic value of WB-MRI in children or young adults, while 10 studies focused on adult patients. In one study, the age of patients was not reported, and we assumed most patients to be adult since 86% (12/14) of patients were diagnosed with diffuse large B-cell lymphoma, a type of non-Hodgkin lymphoma commonly diagnosed at age over 60 (26). Five studies obtained WB-MRI with a magnetic field of 3.0 Tesla, and the remaining 9 used 1.5 Tesla. WB-DWI was applied in 10 of the 14 studies. Contrast-enhanced WB-MRI was applied in 5 studies. Eleven of the 14 studies were prospectively designed. The calculation of concordance rate was available in 4 studies. Direct comparison of the diagnostic performance between WB-MRI and PET/CT was available for 6 studies.

**Table 1:** Basic characteristics of the included studies.

Author/year	Ref.	N	Age	Reference standard	Type of cancer	Magnetic field (T)	WB-DWI	Contrast enhancement	Study design
Chieh et al./2010	(12)	30	58	IUR criteria for MM	Multiple myeloma	3	No	Yes	P
Chieh* et al./2010	(13)	15	48	Revised IWG criteria	Lymphoma	3	Yes	No	P
Marius et al./2011	(14)	12	61	IMWG	Multiple myeloma	3	Yes	No	P
Suzanne et al./2012	(15)	51	14	PET/CT-based criteria	Lymphoma	3	Yes	No	P
Thorsten et al./2012	(16)	31	55	EBMT modified by IUR criteria for MM	Multiple myeloma	3	No	Yes	R
Giuseppe et al./2012	(17)	29	44-83	IMWG	Multiple myeloma	3	No	No	P
Katja et al./2012	(18)	14	NR	Revised IWG criteria	Lymphoma	1.5	Yes	No	P
Sharon et al./2014	(19)	26	61	IMWG	Multiple myeloma	3	Yes	No	P
Marius et al./2015	(20)	64	56	IHP criteria of the IWG for PET/CT	Lymphoma	1.5	Yes	No	P
Arash et al./2018	(21)	38	16	PET/CT-based criteria	Lymphoma	3	Yes	Yes	P
Mohammad et al./2018	(22)	22	62	IMWG	Multiple myeloma	1.5	No	No	P
Ho et al./2020	(23)	42	60	IMWG	Multiple myeloma	1.5	Yes	Yes	R
Ashok et al./2020	(24)	56	15	PET/MRI for lymphoma; PER-CIST and PET/MRI for sarcoma	Lymphoma and Sarcoma	1.5	Yes	Yes	P
Paternain et al. /2020	(25)	27	58	IMWG	Multiple myeloma	3	Yes	No	R

EBMT, European Group for Blood and Marrow Transplantation; IHP, International Harmonization Project; IMWG, International Myeloma Working Group; IUR, international uniform response; IWG, International Working Group; N, number (of patients); NR, not reported; P, prospective; PER-CIST, PET response criteria in solid tumors; R, retrospective; Ref., reference; T, tesla; WB-MRI, whole-body MRI.

### Quality Assessment, Heterogeneity, and Publication Bias

**Figure 2:**
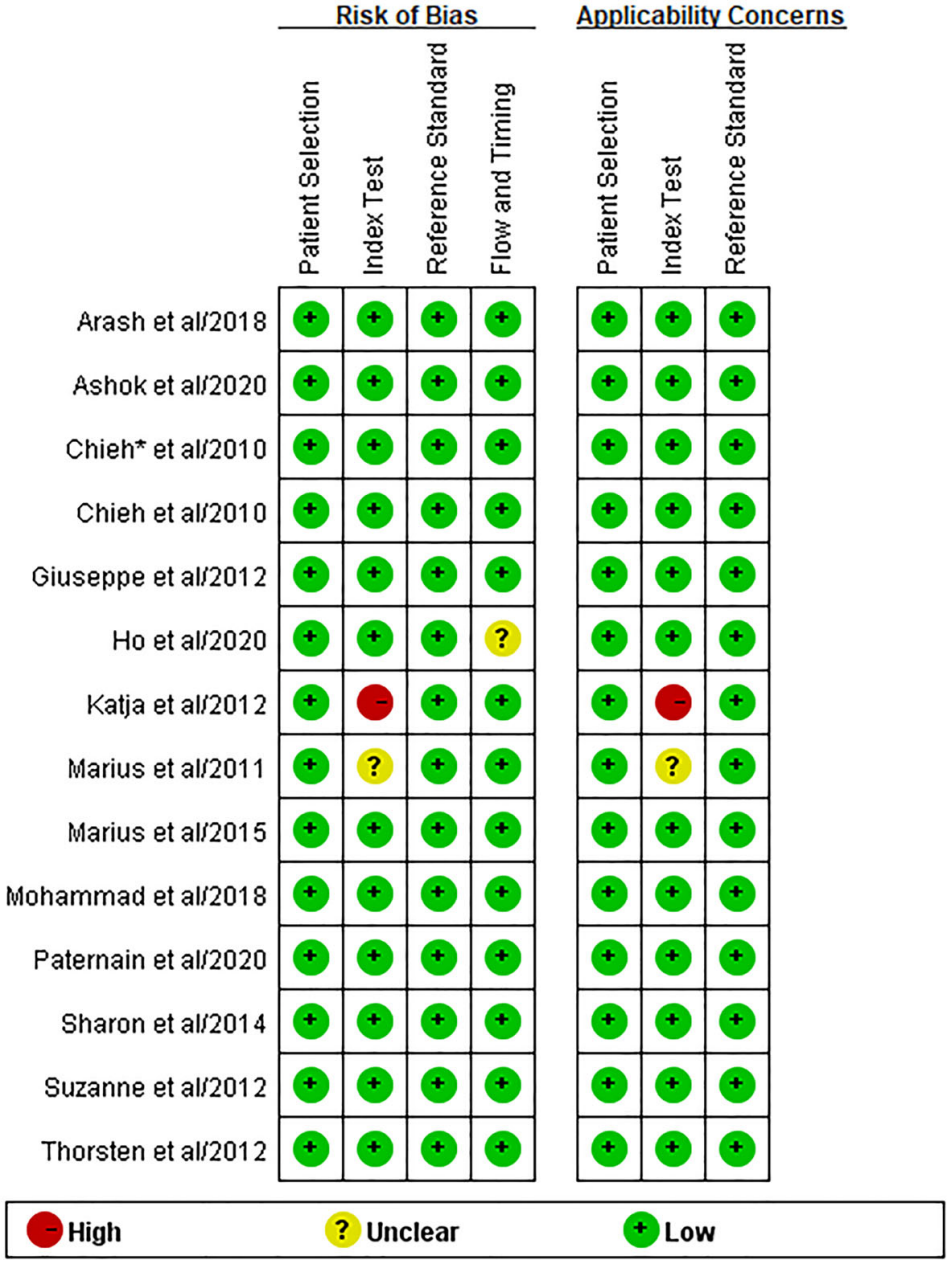
Quality assessment of the included studies using the Quality Assessment of Diagnostic Accuracy Studies (QUADAS-2).

**Figure 2** demonstrates the overall quality of the included studies. The majority of the studies had high quality with a low risk of bias. One study had a high risk of bias and high concern of applicability for the index test since the threshold for the apparent diffusion coefficient (ADC) value to differentiate treatment response was not pre-specific. One study had an unclear risk of bias and concern of applicability for the index test since it did not indicate whether the WB-MRI was interpreted without knowledge of the results of the reference standard. The risk of bias for flow and timing was unclear for one study since it did not provide the interval between WB-MRI and the reference standard.

**Figure 3:**
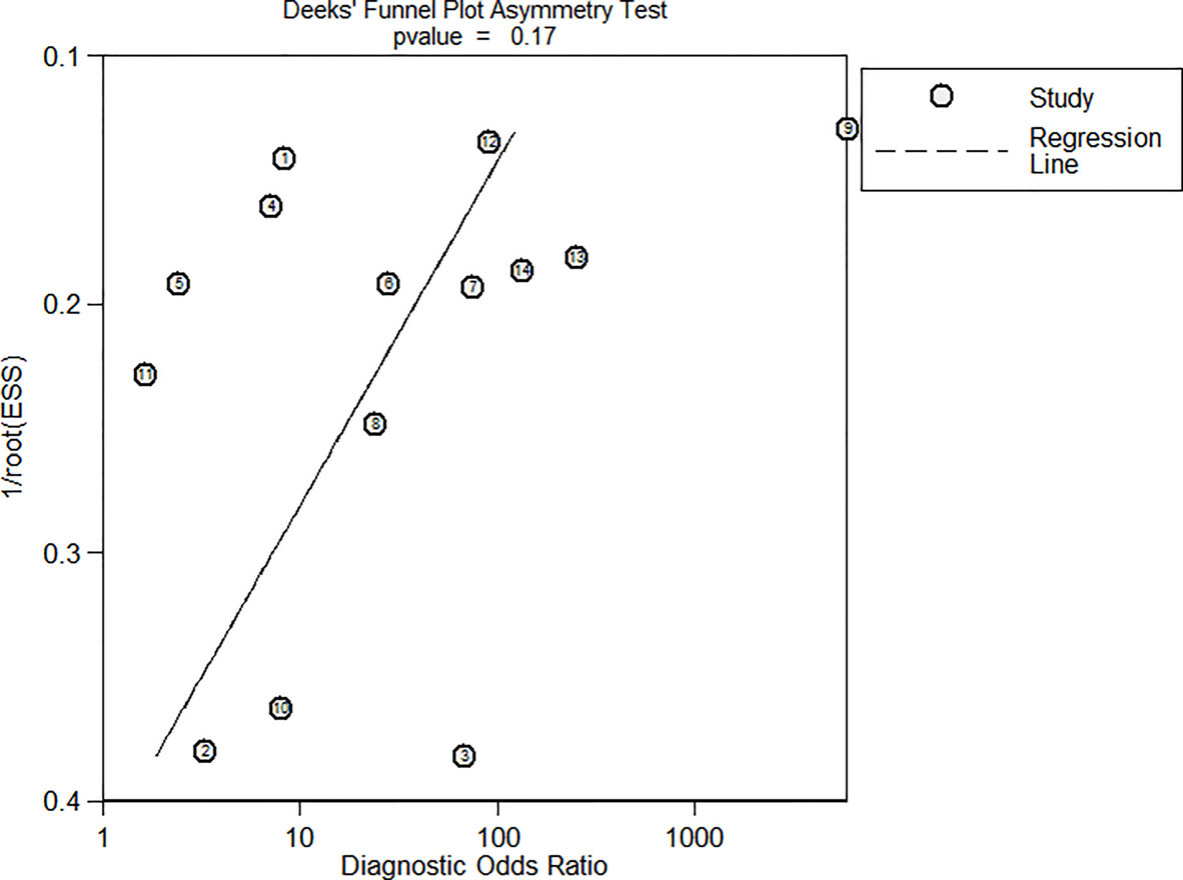
Deek’s funnel plot.

Significant heterogeneity was found for the sensitivity (Q = 98.83, I^2^ = 86.85, p < 0.001) and specificity (Q = 80.81, I^2^ = 83.91, p < 0.001). However, no threshold effect was identified (proportion of heterogeneity likely due to threshold effect = 0.08). **Figure 3** is Deek’s funnel plot, which shows no publication bias (p = 0.17) among the included studies.

### Diagnostic Performance of Whole-Body MRI

**Figure 4:**
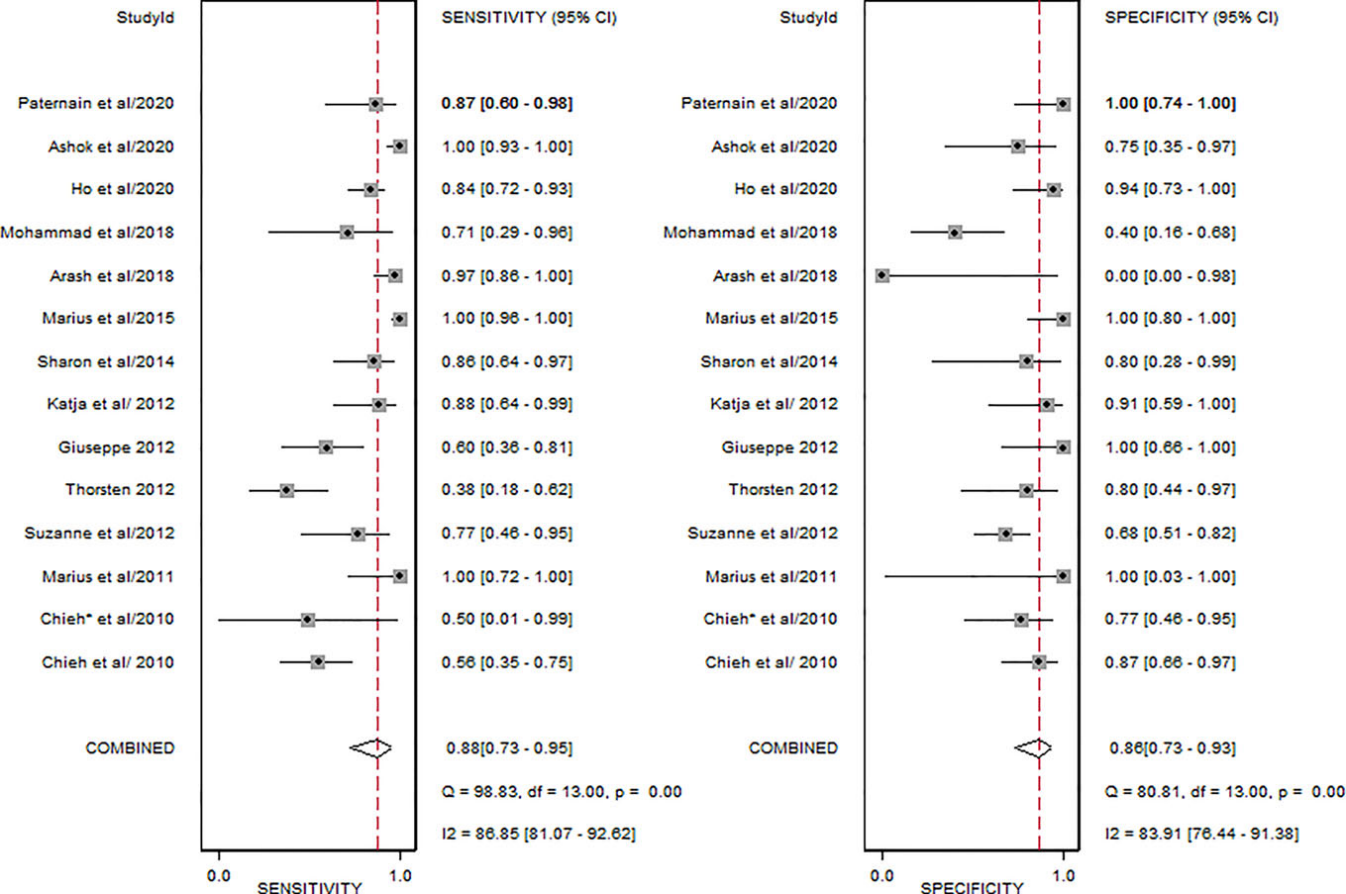
Forest plot of the pooled sensitivity and specificity of the included studies.

**Figure 5:**
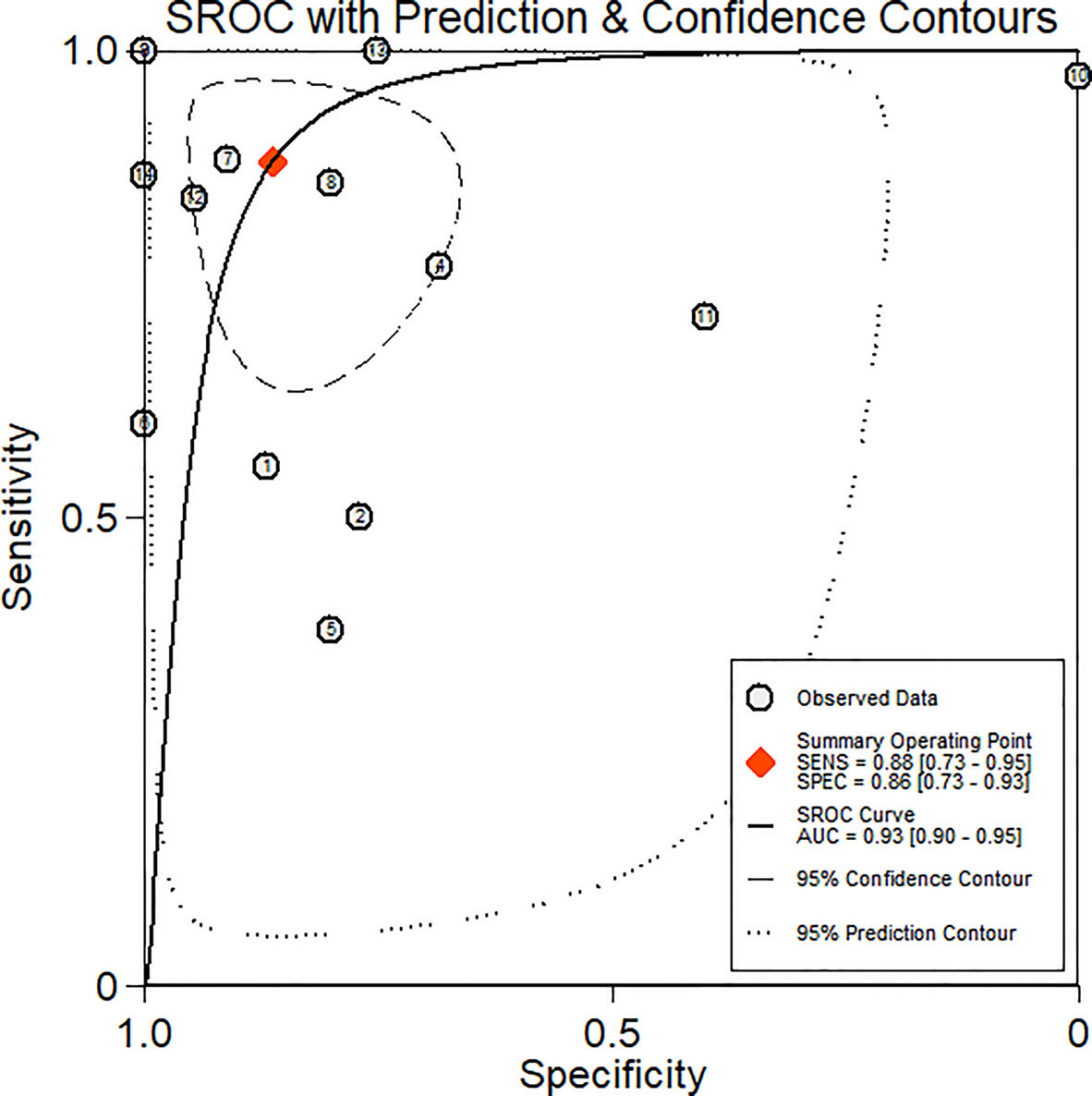
Summary receiver operating characteristics curve.

**Figure 4** shows the forest plots of the sensitivity and specificity for WB-MRI to assess cancer treatment response. The pooled sensitivity, specificity, positive likelihood ratio, and negative likelihood ratio of WB-MRI for cancer therapeutic response assessment were 0.88 (95% CI: 0.73–0.95), 0.86 (95% CI: 0.73–0.93), 6.4 (95% CI: 3.1–13.4), and 0.14 (95% CI: 0.06–0.35), respectively. The SROCs are demonstrated in **Figure 5** with an area under the curve (AUC) of 0.93 (95% CI: 0.90–0.95).

### Subgroup Analysis and Meta-Regression

**Table 2** represents the result of subgroup analysis and meta-regression analysis between each group. In univariate meta-regression, the sensitivity of WB-MRI to assess treatment response was significantly higher for studies in patients with lymphoma and sarcoma than in patients with multiple myeloma (0.96 vs. 0.76, p < 0.001). In addition, the sensitivity was significantly higher for studies using WB-MRI with WB-DWI than those that did not (0.94 vs. 0.55, p = 0.02). However, when performing multivariate meta-regression, WB-DWI was the only factor related to the heterogeneity (p = 0.012).

**Table 2:** Subgroup analysis and meta-regression.

Parameter	No. of studies	Sensitivity	p (univariate meta-regression)	p (multivariate meta-regression)	Specificity	p (univariate meta-regression)
Patient population			0.19	/		0.17
Children and young adults	3	0.97 (0.90, 1.00)			0.70 (0.35, 1.00)	
Adults	11	0.83 (0.68, 0.97)			0.90 (0.81, 0.99)	
Type of cancer			<0.001	0.72		0.94
Multiple myeloma	8	0.76 (0.58, 0.93)			0.89 (0.78, 1.00)	
Lymphoma or sarcoma	6	0.96 (0.91, 1.00)			0.84 (0.69, 1.00)	
Magnetic field			0.07	/		0.67
1.5 T	9	0.80 (0.72, 0.97)			0.86 (0.74, 0.98)	
3.0 T	5	0.95 (0.89, 1.00)			0.86 (0.72, 1.00)	
WB-DWI			0.02	0.012		0.80
Applied	10	0.94 (0.89, 0.98)			0.88 (0.78, 0.98)	
Not applied	4	0.55 (0.31, 0.79)			0.80 (0.60, 1.00)	
Contrast enhancement						
Applied	5	0.86 (0.68, 1.00)	0.86	/	0.85 (0.67, 1.00)	0.61
Not applied	9	0.89 (0.77, 1.00)			0.87 (0.75, 1.00)	
Study design			0.18	/		0.19
Prospective	11	0.91 (0.81, 1.00)			0.83 (0.71, 0.95)	
Retrospective	3	0.74 (0.39, 1.00)			0.94 (0.84, 1.00)	

WB-MRI, whole-body MRI.

### Concordance Rate of Whole-Body MRI

**Figure 6:**
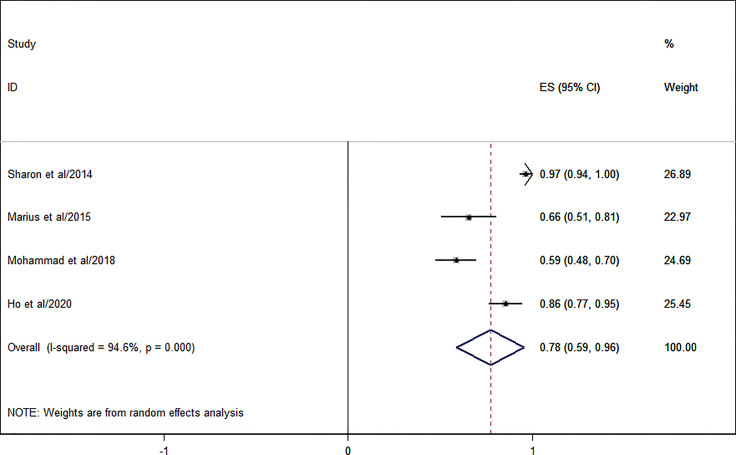
Forest plot of the pooled concordance rate for whole-body MRI (WB-MRI) compared to reference standard.

Four studies with a total number of 200 patients and 271 patients’ follow-up were available for the concordance rate. **Figure 6** shows the forest plot of the concordance rate of WB-MRI. The pooled concordance rate of WB-MRI compared with the reference standard was 0.78 (95% CI: 0.59–0.96).

### Whole-Body MRI Versus PET/CT for Cancer Treatment Response Assessment and Contrast-Enhanced Versus No Contrast-Enhanced Whole-Body MRI for Lymphoma Treatment Response Assessment

**Table 3** shows the specific subgroup analysis. Six studies with a total patient number of 179 and 193 patient follow-up were available for the comparison of the diagnostic performance between WB-MRI and PET/CT for cancer treatment assessment. There was no significant difference either for the sensitivity (0.83 vs. 0.92, p = 0.11) or for the specificity (0.87 vs. 0.76, p = 0.73). For treatment assessment of lymphoma, contrast-enhanced WB-MRI was applied in 2 studies, while the rest of the 4 studies were conducted without contrast-enhanced sequences. Contrast-enhanced WB-MRI had a higher sensitivity (0.99 vs. 0.79, p < 0.001) but a similar specificity (0.62 vs. 0.72, p = 0.76) for lymphoma treatment assessment as compared to non-enhanced WB-MRI.

**Table 3:** Comparison of specific subgroups.

Examination	Number of studies	Sensitivity	p	Specificity	p
Whole-body MRI vs. PET/CT					
Whole-body MRI	6	0.83 (0.54, 0.95)	0.11	0.87 (0.60, 0.97)	0.73
PET/CT		0.92 (0.78, 0.98)		0.76 (0.62, 0.86)	
Contrast-enhanced vs. non-enhanced for lymphoma					
With contrast enhancement	6	0.99 (0.97, 1.00)	<0.001	0.62 (0.19, 1.00)	0.76
Without contrast enhancement		0.79 (0.96, 0.93)		0.70 (0.52, 0.87)	

## Discussion

The result of the present study showed that WB-MRI had a relatively high sensitivity (0.88) and specificity (0.86) for therapeutic response assessment in patients with lymphoma, multiple myeloma, and sarcoma. The AUC of the SROCs was high (0.93). In addition, we compared the diagnostic performance of WB-MR with PET/CT, which was commonly used as the reference standard for treatment response assessment for many types of malignancy (27, 28), and we found that there was no significant difference in the sensitivity and specificity. Since the currently used guideline would subclassify the treatment response into CR, PR, SD, and PD (29), we calculated the pooled concordance rate of WB-MRI compared with the reference standard within a subgroup of the studies, which revealed a moderately high concordance rate of 78%. These indicate that WB-MRI is an effective imaging modality for assessing treatment response in patients with lymphoma, multiple myeloma, and sarcoma.

As there was significant heterogeneity for the pooled sensitivity and specificity, subgroup analysis and meta-regression were performed. Studies with WB-DWI showed a significantly higher sensitivity for cancer treatment assessment than those without (0.94 vs. 0.55, p = 0.02 at univariate meta-regression and p = 0.012 at multivariate meta-regression). This is not surprising since DWI is a multipotent imaging modality for cancer diagnosis (30), staging (31), and treatment response assessment (32). Previous studies showed that ADC value increased significantly in responders compared to non-responders (5, 33). Additionally, interim ADC had been shown to help identify non-responder lesions for Hodgkin lymphoma after a few courses of chemotherapy (34). It was believed that the increase of ADC value was due to loss of cellularity caused by effective treatment (35). Therefore, the adding of WB-DWI may increase the sensitivity for the assessment of cancer treatment response. At univariate meta-regression, the sensitivity was significantly higher in patients with lymphoma and sarcoma than patients with multiple myeloma (0.96 vs. 0.76, p < 0.001). However, at WB-DWI adjusted multivariate meta-regression, no significant difference was found (p = 0.72), which revealed that the difference was caused by whether WB-DWI was applied or not. Indeed, of the 6 studies that focused on multiple myeloma, four of them did not obtain WB-DWI for patients. This addressed the importance of WB-DWI in the assessment of cancer treatment response.

The application of contrast-enhanced sequences was not associated with either an increased sensitivity or specificity in the subgroup analysis. However, when we subsequently analyzed WB-MRI for assessment of lymphoma therapeutic response, contrast-enhanced WB-MRI was associated with increased sensitivity (0.99 vs. 0.79, p < 0.001) but similar specificity (0.62 vs. 0.72, p = 0.76) as compared to unenhanced WB-MRI. Currently, the use of contrast media for WB-MRI scanning for lymphoma is still being debated (36–38). Previous studies reported that dynamic contrast-enhanced MRI improved the accuracy for detection of splenic involvement of Hodgkin lymphoma (39). The result of the present study might provide evidence for supporting the use of contrast-enhanced sequences in WB-MRI scanning protocols for the assessment of lymphoma treatment response.

In the field of pediatric oncology, WB-MRI is gaining more and more attention. On the one hand, it imparts a zero dose of ionizing radiation, which is particularly important for children and adolescents since their organs and tissues are more radiosensitive (40). On the other hand, WB-MRI could provide high-quality imaging of the entire body within 1 h (41). The result of the subgroup analysis showed no significant difference in the diagnostic performance for WB-MRI to assess treatment response between the pediatric group and adult group. Of the 3 studies, 2 analyzed pediatric patients with lymphoma, and 1 analyzed pediatric patients with lymphoma (66%) and sarcoma (34%). The result of the present study might provide evidence for the use of WB-MRI in pediatric oncology for the treatment assessment of lymphoma and sarcoma.

Previous meta-analyses have shown that WB-MRI had comparable diagnostic performance with PET/CT for distant malignancies (42) and pretherapeutic staging of lymphoma (43). To the best of our knowledge, this is the first meta-analysis to evaluate the diagnostic performance of WB-MRI for hematological malignancies’ therapeutic response assessment. Chong et al. have conducted a meta-analysis, which found MRI a potential surrogate imaging modality for treatment response assessment in patients with glioblastoma (44). Moreover, MRI has been suggested for rectal cancer response evaluation to neoadjuvant chemoradiation (45). The present meta-analysis showed that WB-MRI, especially combined with WB-DWI, was a promising imaging tool for treatment response assessment in widely disseminated malignancies such as multiple myeloma, lymphoma, and sarcoma. Plus, it had a comparable diagnostic performance with PET/CT. However, there are still some drawbacks of WB-MRI. First, quantitative assessment of treatment response beyond the mere size evaluation on WB-MRI is still challenging. Second, there is no consensus on WB-MRI scanning protocols and ADC value thresholds for the assessment of treatment response (36). Third, WB-MRI has some challenging locations in which a miscalculation of ADC measurements may hamper the correct evaluation of ADC values of lymph nodes, particularly in the mediastinum, which is a very common location of lymphoma (46). In comparison, interim PET/CT has a consolidated prognostic role in hematological malignancies, especially in Hodgkin lymphoma where a PET-driven therapy is routinely accepted and performed using FDG uptake to understand the tumor’s chemosensitivity (37).

There were several limitations of our meta-analysis. First, the included studies had significant heterogeneity while generating the diagnostic parameters, which might decrease the general applicability of the pooled estimates. However, through univariate and multivariate meta-regression, we identified the cause of the heterogeneity and addressed the importance of applying WB-DWI in WB-MRI for cancer treatment response assessment. Second, the composition of the patient population was heterogeneous, with most patients with hematogenic tumors and a small number of patients with sarcoma. But in subgroup analysis and meta-regression, no significant difference was found for the diagnostic performance of WB-MRI for different types of tumors. Third, we only included studies written in English, which might lead to Tower of Babel bias (47).

In conclusion, WB-MRI has good sensitivity and specificity for the evaluation of the treatment response of multiple myeloma, lymphoma, and sarcoma. The adding of WB-DWI would further increase the sensitivity. Additionally, WB-MRI may have comparable performance with PET/CT for therapeutic response assessment, which is particularly attractive in the field of pediatric oncology.

## Data Availability Statement

The original contributions presented in the study are included in the article/**Supplementary Material**. Further inquiries can be directed to the corresponding authors.

## Author Contributions

GL, YG, and HZ designed the study. GL, XZ, YL, and WT performed the literature search. GL and XZ reviewed the articles. WS and SZ acquired the data. YL, WT, WS, and SZ performed the statistical analysis. GL, YG, and HZ drafted the manuscript. All authors revised and approved the final version of the manuscript.

## Funding

This study has received funding from the Sanming Project of Medicine in Shenzhen (No. SZSM202011005).

## Conflict of Interest

The authors declare that the research was conducted in the absence of any commercial or financial relationships that could be construed as a potential conflict of interest.

## Publisher’s Note

All claims expressed in this article are solely those of the authors and do not necessarily represent those of their affiliated organizations, or those of the publisher, the editors and the reviewers. Any product that may be evaluated in this article, or claim that may be made by its manufacturer, is not guaranteed or endorsed by the publisher.

## Abbreviations

CR, complete response; FN, false negative; FP, false positive; PD, progressive disease; PR, partial response; QUADAS-2, Quality Assessment of Diagnostic Accuracy Studies; SD, stable disease; SROCs, summary receiver operating characteristic curves; TN, true negative; TP, true positive; WB-MRI, whole-body magnetic resonance imaging; WB-DWI, whole-body diffusion-weighted imaging.
