# Supplementary material for: Whole-Body MRI Is an Effective Imaging Modality for Hematological Malignancy Treatment Response Assessment: A Systematic Review and Meta-Analysis
Source: Front Oncol. 2022 Feb 18;12:827777. doi: 10.3389/fonc.2022.827777 (PMC8894650; doi:10.3389/fonc.2022.827777)
Supplement: Supplementary file 1 [file Table_1.docx]

PubMed: (‘‘whole body [Magnetic Resonance Imaging](https://www.ncbi.nlm.nih.gov/mesh/68008279)’’[Mesh] OR whole body MR imaging [tiab] OR whole body MRI[tiab] OR WB-MRI[tiab] OR whole body diffusion magnetic resonance imaging[tiab] OR whole body diffusion MRI[tiab] OR whole body diffusion weighted MRI[tiab] OR WB-DWI[tiab] OR Whole body[tiab]) AND (treatment response[tiab] OR therapeutic response[tiab] OR response assessment[tiab])

Embase: ('whole body magnetic resonance imaging' OR 'whole body MR imaging' OR 'whole body MRI' OR 'WB-MRI' OR 'whole body diffusion magnetic resonance imaging' OR 'whole body diffusion MRI' OR 'whole body diffusion weighted MRI' OR 'WB-DWI') AND ('treatment response' OR 'therapeutic response' OR 'response assessment')

Web of Science: (Ts=(whole body Magnetic Resonance Imaging) OR TI=(whole body MR imaging OR whole body MRI OR WB-MRI OR whole body diffusion magnetic resonance imaging OR whole body diffusion MRI OR whole body diffusion weighted MRI OR WB-DWI OR Whole body)) AND TI=(treatment response OR therapeutic response OR response assessment)
